# Genetic improvement of clubroot resistance in the recessive genic male sterile line YA001 in *Brassica napus*

**DOI:** 10.3389/fpls.2025.1726940

**Published:** 2025-12-16

**Authors:** Yingfen Jiang, Weixin Fei, Xingjie Wu, Fengxiang Chen, Wenjing Li, Ying Wang, Lanlan Zhan, Cheng Li

**Affiliations:** 1 Institute of Crop Research, Anhui Academy of Agricultural Sciences/Key Laboratory of Crop Quality Improvement of Anhui Province, Hefei, Anhui, China; 2 Key Laboratory of Biology and Genetic Improvement of Oil Crops, Ministry of Agriculture and Rural Affairs, Wuhan, China; 3 Wuhan Agricultural Technology Extension Center, Wuhan, China

**Keywords:** *Brassica napus*, clubroot disease, recessive genic male-sterile, marker assistant selection, CRb

## Abstract

Clubroot disease poses a serious challenge to global rapeseed production, including major growing regions in China. Introgressing clubroot-resistant (CR) genes into rapeseed is an efficient way to prevent the spread of this disease. YA001 is a stable recessive genic male-sterile (RGMS) three-line system in *Brassica napus* with good combining ability but high susceptibility to clubroot. To improve the clubroot-resistant of YA001, ‘Bing409R’, which exhibits excellent disease resistance and contains the CR gene *CRb*, was used as the donor parent, and the male-sterile line YA001 was used as the female parent for hybridization in Anhui Province. The temporary maintainer line of YA001 was then used as the recurrent parent for backcrossing. Marker assistant selection was conducted for the CR gene and fertility-related genes. In each backcrossing generation, individuals with the genotype *RrMs_3_ms_3_Rf^b^Rf^c^* were selected. After 4 generations of backcrossing, self-pollination was performed, and homozygous CR RGMS three-lines were developed from the self-pollinated progeny. Inoculation tests under controlled conditions showed that the disease index of improved YA001 was 8.7, exhibited high clubroot resistance. Hybrid combinations developed by CR YA001 demonstrated superior yield performance and effective disease resistance when cultivated in infested region. And the highest-yielding of CR combinations achieved 3273.15 kg/ha while susceptible control was only 1279.8 kg/ha. This study provides a rapidand efficient method for breeding homogeneous CR RGMS three-lines. Furthermore, these developed lines serve as an important genetic resource for the high-throughput selection of strong heterosis and CR combinations, thereby contributing to the control of clubroot spread.

## 1 Introduction

Rapeseed is the largest oilseed crop and the second most important winter crop in China, with an annual cultivation area of approximately 7.0 million hectares. It serves as the primary domestic source of edible vegetable oil and a significant provider of feed protein (Liu et al., 2019). In recent years, clubroot disease has rapidly spread across rapeseed-growing regions in China affecting approximately 1.33 million hectares and posing a serious threat to the industry (Wang et al., 2021). This disease, caused by the protist *Plasmodiophora brassicae*, induces gall formation after roots infected, impairing nutrient and water uptake. Severe infections can lead to complete plant wilting and total yield loss in heavily affected fields (Hwang et al., 2012; Chai et al., 2014).

As a soil-borne pathogen, clubroot produces resting spores that can persist in soil for more than a decade (Dixon, 2009). Combined with the obligate parasitic nature of *P. brassicae*, this disease is hard to be removed from the soil, presenting a substantial threat to rapeseed production in China and worldwide (Askarian et al., 2021). Previous experiences indicated that breeding and cultivating resistant varieties stands as the most cost-effective and sustainable way (Yu et al., 2021). *P. brassicae* exhibits physiological race differentiation, and correspondingly, its resistance genes demonstrate race-specific effectiveness (Neik et al., 2017). The primary sources of clubroot resistance in *Brassica* species originate from European turnips (*Brassica rapa*). To date, 28 resistance loci or QTLs have been mapped, among which *CRa/CRb/Rcr1* and *Crr1a/CRA8.2.4* have been successfully cloned and functionally validated (Matsumoto et al., 1998; Suwabe et al., 2003; Hirai et al., 2004; Piao et al., 2004; Sakamoto et al., 2008; Ueno et al., 2012; Chen et al., 2013; Hatakeyama et al., 2013; Chu et al., 2014; Yu et al., 2017, 2021; Huang et al., 2017, 2019; Hirani et al., 2018; Pang et al., 2018; Laila et al., 2019; Fredua-Agyeman et al., 2020; Karim et al., 2020; Rahaman et al., 2022; Yang et al., 2022; Hu et al., 2024; Lai et al., 2025). All of which encode a toll-Intereleukin-1 receptor/nucleotide binding site/leucine-rich repeat (TIR-NBS-LRR, TNL) protein (Eitas and Dangl, 2010; Yang et al., 2022). However, *Brassica napus* possesses extremely limited resistance sources, and the majority of clubroot resistant genes in *B. napus* have been derived from *B. rapa* (Dakouri et al., 2018). Interspecific hybridization remains the most effective approach for introgressing clubroot resistance genes from different sources into rapeseed. Recently, the research team at Huazhong Agricultural University successfully developed the clubroot resistant (CR) rapeseed line ‘Bing409R’ by introgressing the *CRb* resistance locus from the donor parent Chinese cabbage (*Brassica rapa*) ‘CR Shinki’ into the elite *Brassica napus* paternal line ‘Bing409’ (Hatakeyama et al., 2017). The resulting ‘Bing409R’ is regarded as an excellent resistance germplasm since it exhibits immune-level resistance against multiple physiological races of *P. brassicae* prevalent across Chinese epidemic regions, thus it has been widely used in the field (Li et al., 2021).

Recessive genic male sterility (RGMS) three-line system in *Brassica napus* is an important approach for utilizing heterosis in rapeseed. With its stable sterility and flexible combination, this system has been widely employed in rapeseed hybrid breeding (Chen and Hu, 1998a). The sterility of RGMS is controlled by the interaction between one sterility gene pair (*Ms_3_/ms_3_*) and one tri-allelic gene (*Rf^a^/Rf^b^/Rf^c^*). *Ms_3_* is the wild-type fertile gene, while its recessive allele *ms_3_* is the sterile mutant. The *Rf* locus contains three alleles: *Rf^a^* (*Ms_4_*), *Rf^b^* (*Rf*), and *Rf^c^* (*rf*). Among them, *Rf^c^* is the wild-type fertile gene, *Rf^a^* is the mutant restorer fertile gene, and *Rf^b^* is the mutant sterile gene. Their dominance relationship follows: *Rf^a^* > *Rf^b^* > *Rf^c^.* Male sterility occurs when plants are homozygous recessive for *ms_3_ (ms_3_ms_3_*) and carry either *Rf^b^Rf^b^* or *Rf^b^Rf^c^* genotypes at the *Rf* locus. All other genotypic combinations result in normal male fertility. In naturally growing rapeseed populations, the wild-type genotype *Ms_3_Ms_3_Rf^c^Rf^c^* accounts for the vast majority, with very rare occurrences of *Ms_3_Ms_3_Rf^b^Rf^b^* and *Ms_3_Ms_3_Rf^a^Rf^a^* genotypes, which both being mutant forms of *Rf^c^* (Dong et al., 2010; 2012). The RGMS three-line system consists of: two-type line (A line, sterile, *ms_3_ms_3_Rf^b^Rf^b^* and B line, fertile, *Ms_3_ms_3_Rf^b^Rf^b^*), and the temporary maintainer line (TM line, fertile *ms_3_ms_3_Rf^c^Rf^c^*).The A line crossed to the TM line to produce completely sterile lines (CS line, *ms_3_ms_3_Rf^b^Rf^c^*). The CS lines are then crossed with restorer lines to produce commercial F_1_ hybrid seeds (Dong et al., 2014).

YA001 is an elite improved line derived from RGMS ‘9012A’ in *Brassica napus*, characterized by its stable sterility, good combining ability and high oil content. Using this line, a series of high-yielding and high-quality hybrid cultivars have been registered and released (Chen et al., 2002), but YA001 remains highly susceptible to clubroot disease. Enhancing clubroot resistance in RGMS line of YA001 is essential to counter the detrimental effects of the rapidly spreading clubroot disease on rapeseed production. In this study, to improve the clubroot-resistant of YA001, the CR material ‘Bing409R’ was used as the donor parent to transfer the dominant resistance locus *CRb* into YA001, aiming to create CR homogeneous RGMS three-line system. The results will establish an important foundation for high-throughput breeding of superior CR rapeseed cultivars and provide essential genetic resources for mitigating the shortage of CR cultivars in rapeseed production in China.

## 2 Materials and methods

### 2.1 Plant materials and pathogen isolates

‘Bing409R’ was used as the donor parent with double low seeds quality (low erucic acid and low glucosinolate), which carries the CR gene *CRb* (designated as *R*). YA001 is a semi-winter type, double-low RGMS line of *Brassica napus*. The plants are of medium height (~150 cm), with abundant branching and high pod numbers, an oil content of 44%–48%, and strong cold tolerance. The clubroot pathogen used for inoculation was collected from Huangshan region in Anhui Province, which belongs to the predominant physiological race 4.

### 2.2 Research methods

#### 2.2.1 Experimental procedure

Using ‘Bing409R’ as the paternal parent and YA001 as the maternal parent, hybridization was performed in Anhui province. The resulting F_1_ generation exhibited heterozygous genotypes (*RrMs_3_ms_3_Rf^b^Rf^c^*) at all loci without selection. Selected vigorous individual plants were backcrossed with the TM line (*rrms_3_ms_3_Rf^c^Rf^c^*). Beginning with the BC_1_ generation, molecular marker-assisted selection (MAS) was performed to select target fertility-related genes (*Ms_3_/ms_3_* and *Rf^b^/rf^c^*) and clubroot resistance gene along with fast screening double-low quality traits. Primer sequences and PCR reaction conditions are provided in **Table 1**.

**Table 1:** Primer pairs for molecular marker assistant selection.

Primer name	Forward primer (5’—3’)	Reverse primer (5’—3’)	Weight Size (bp)
A3-7	CCTTACAAAGCTCATATACTT	AGCCATCGTCCACAACTCG	160
Pms	GGATTTAGTGATGCAACACATG	TGCGTATTCATCTGGTTCATCA	200
A7-Rf	CGGCCATTAGATAGGGCATT	ACGATCCCAATCAGCTCAAC	250

**Figure 1:**
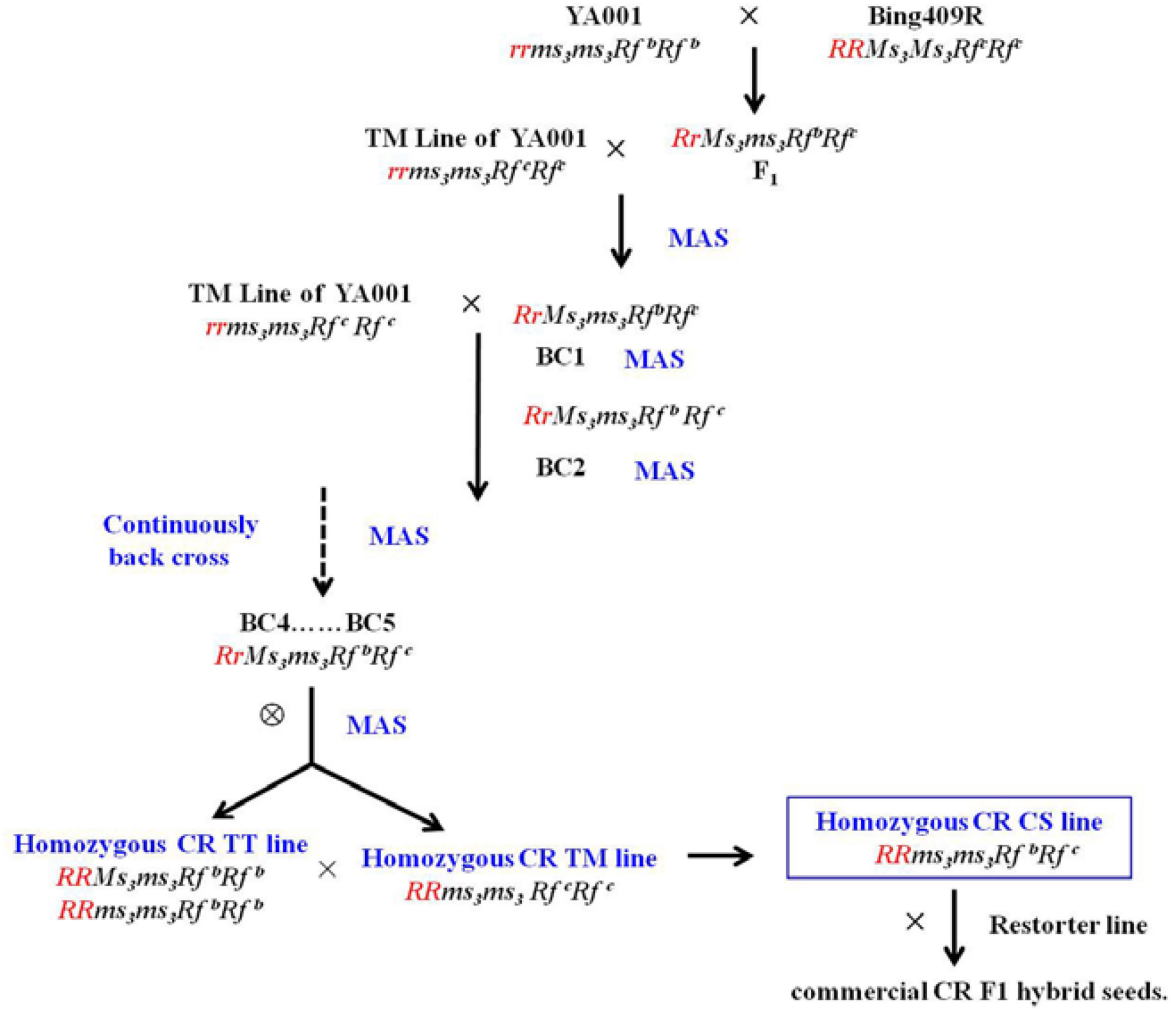
Experimental flowchart.

Backcrossing continued until the BC_4_-BC_5_ generations, and then background recovery rate was assessed using the SNP panel. For plants achieving over 90% background recovery, individuals with the tri-heterozygous genotype (*RrMs_3_ms_3_Rf^b^Rf^c^*) were selected for self-pollination. Molecular markers were used to select CR two-type lines (*RRMs_3_ms_3_Rf^b^Rf^b^* + *RRms_3_ms_3_Rf^b^Rf^b^*) and CR TM line (*RRms_3_ms_3_Rf^c^Rf^c^*). The complete technical route is illustrated as in **Figure 1**.

#### 2.2.2 Clubroot resistance evaluation

##### 2.2.2.1 Inoculation of clubroot pathogen under controlled conditions

The indoor inoculation for clubroot resistance assessment was conducted using the greenhouse root-drenching method (Hwang et al., 2011). Briefly, clubroot galls stored at -20 °C were thawed to room temperature and softened. The galls were homogenized in a blender at a ratio of 30 g galls per 100 mL distilled water. The homogenate was filtered and centrifuged to remove the supernatant. The number of resting spores was counted under a microscope, and the spore concentration was adjusted to 1×10^7^ spores/mL for inoculation. Sterilized nutrient substrate was used for seedling cultivation. Plug trays filled with the substrate were thoroughly saturated with tap water. A ~2 cm deep hole was made in the center of each cell using a pencil, and seeds were sown into the holes. Subsequently, 1mL of the prepared spore suspension was pipetted into each hole. The inoculated trays were incubated under controlled conditions: light intensity of 200 μmol·m^-^²·s^-^¹, 14/10 h (light/dark) photoperiod, and 25/20 °C (day/night) temperature. After 42 days post-inoculation (dpi), roots were gently washed, and disease phenotypes were evaluated.

##### 2.2.2.2 Clubroot disease rating

The disease evaluation was assessed 6 weeks after inoculation on a 0–3 scale (Strelkov et al., 2006), with disease severity graded based on gall size and distribution: Where, 0 Grade=No visible galls; 1 Grade=No galls on main root, few small galls on lateral roots; 2 Grade=Medium-sized galls on both main root and lateral roots; 3 Grade=Gall diameter/length exceeding 3× stem base diameter, or presence of ulcerated galls. Disease parameters: Disease incidence (%) = (Number of infected plants/Total plants assessed) × 100; Disease index = [∑a Number of diseased plants × grade value)/(Total plants assessed × maximum grade)] × 100. Resistance evaluation criteria (based on disease index): Immune (I): Disease index = 0, Highly resistant (HR): 0 < Disease index ≤ 10, Resistant (R): 10 < Disease index ≤ 30, Susceptible (S): 30 < Disease index ≤ 50, Highly susceptible (HS): Disease index > 50.

#### 2.2.3 Quality analysis

For seeds harvested from each backcross generation, quality analysis was performed using a near-infrared spectrometer (NIRS DS2500). Individuals failing to meet the double-low quality standards were eliminated, while qualified double-low seeds were retained for planting in the following year.

#### 2.2.4 Molecular marker screening

Genomic DNA was extracted from young leaves of parental lines and individual plants of each generation using the CTAB rapid extraction method (Murray and Thompson, 1980). The PCR was performed in a 10 µL reaction system containing 100 ng DNA templates, 250 nmol L^-1^ of each forward and reverse primer, 0.25 nmol L^-1^ dNTPs, and 1 U µL^-1^ Taq DNA polymerase.

The markers used for detection have been previously validated in earlier experiments as clear, stable, and reliable tightly linked markers. The following marker systems were employed: fertility-related gene *Ms_3_/ms_3_*, was amplified using co-dominant marker Pms (annealing at 60 °C for 30 s, extension at 72 °C for 30 s); The *Rf* gene was screened using marker A7-Rf (annealing at 50 °C for 30 s, extension at 72 °C for 30 s), while the CR gene was detected with marker A3-7 (annealing at 55 °C for 30 s, extension at 72 °C for 30 s). Then PCR products were separated on 6% denaturing polyacrylamide gels(PAGE)at constant power (70W) for 45 minutes, followed by silver staining, development, and band pattern analysis. Primer sequences are detailed in **Table 1**.

#### 2.2.5 SNP genotyping and genetic similarity calculation

Genome-wide genotyping was performed using the Illumina CropSNP 50K array (Thomson et al., 2017). Briefly, 200ng of DNA per sample was enzymatically fragmented, hybridized to SNP probes, amplified, and fluorescently labeled. Raw intensity data were processed with GenomeStudio™ Software (v2.0) for genotype calling. Genetic similarity was estimated based on Identity-by-State (IBS) using high-quality autosomal SNPs. The similarity coefficient (S) between two individuals was calculated as: S = (Number of identical alleles)/(2 × Total SNPs).

## 3 Results and analysis

### 3.1 Genotype selection in backcross generations

Beginning with the BC_1_ generation derived from backcrossing with the TM line, each generation was subjected to molecular marker screening for the *RrMs_3_ms_3_Rf^b^Rf^c^* genotype. According to Mendelian inheritance patterns in backcross populations, the expected ratio of disease-resistant genotypes is 50% (50% *Rr* + 50% *rr*).

**Figure 2:**
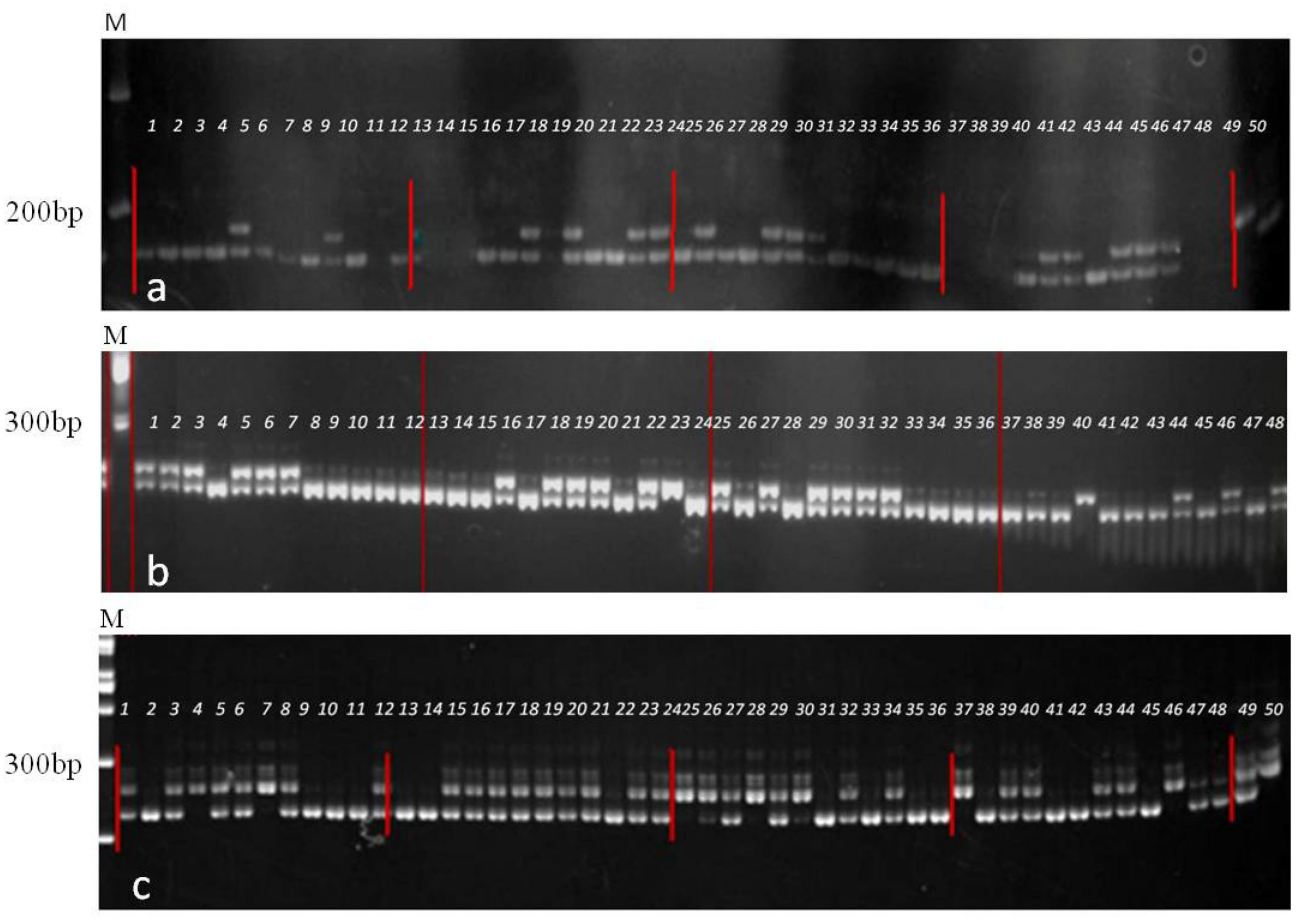
Electrophoresis map of molecular marker-assisted selection. **(a)** shows the PCR amplification electrophoretogram of the co-dominant marker A3–7 for clubroot resistance in the BC_1_ population. Lanes 1–46 correspond to individual plants being tested, Lanes 49 and50 represent the resistant (*RR*) and susceptible (*rr*) controls, respectively. The higher molecular weight band represents the disease-resistant specific band. **(b)** shows the PCR amplification electrophoretogram of the RGMS allele locus *Ms3/ms3* in the segregation population BC_1_ with marker Pms. The higher molecular weight band corresponds to the sterile genotype *ms_3_ms_3_*, lanes 1–46 represent individual plants under genotyping, Lanes 47 and 48 serve as control samples for *Ms_3_Ms_3_* and *Ms_3_ms_3_* genotypes, respectively. **(c)** shows the PCR amplification electrophoretogram of the RGMS allele locus *Rf^b^/Rf^c^* in the segregation population BC_4_F_1_ with marker A7-Rf, Lanes 1–48 represent individual plants undergoing genotyping, Lanes 49 and 50 serve as control samples for *Rf^b^Rf^c^* and *Rf^c^ Rf^c^* genotypes, respectively. M: DNA marker.

As shown in **Table 2**, the genotypic analysis of BC_1_ progenies from the line M205 was performed. The results indicated that about 44 out of the 87 screened plants carried the R resistance gene (chi-square (χ²) = 0.0115), which met the expected 1:1 Mendelian ratio (p>0.05) (**Figure 2a**).

**Table 2:** Genotype distribution of BC_1_ progeny of the line M205.

Line number	Genotype	Number of individuals	Fertility
1	*RrMs_3_ms_3_Rf^b^Rf^c^*	11	fertile
2	*RrM_3_ms_3_Rf^c^Rf^c^*	9	fertile
3	*Rrms_3_ms_3_Rf^c^ Rf^c^*	10	fertile
4	*Rrms_3_ms_3_Rf^b^Rf^c^*	10	sterile
5	*rrMs_3_ms_3_Rf^b^Rf^c^*	10	fertile
6	*rrMs_3_ms_3_Rf^c^Rf^c^*	8	fertile
7	*rrms_3_ms_3_Rf^c^ Rf^c^*	9	fertile
8	*rrms_3_ms_3_Rf^b^Rf^c^*	11	sterile
Total		87	

Fertility-related genes were also screened during the process. Since backcrossing was performed with the TM line, all loci segregated in a 1:1 ratio for *RrMs_3_ms_3_Rf^b^Rf^c^* and *rrms_3_ms_3_Rf^c^Rf^c^*, theoretically producing eight possible genotype combinations, each with an equal probability of 1/8. As shown in **Table 2** for the line M205: fertile and sterile genotypes each accounted for 50% of the population and every genotype represents the same 1/8 ratio (χ² value: 0.768< Critical χ² value14.067), which met the expected ratio. To ensure the transmission of both fertility-related genes and CR gene in each generation, only plants with the *RrMs_3_ms_3_Rf^b^Rf^c^* genotype were selected for backcrossing with the TM line. In practice, selection of backcross plants was based on comprehensive traits including agronomic performance and quality characteristics. Additionally, to prevent losses due to pests, diseases, or field management practices, a minimum of 10 selected plants must be retained at each generation. This necessitated a minimum population size of approximately 80 plants per backcross generation to statistically ensure the availability of target genotypes.

### 3.2 Selection of CR genic male sterile three-line system

After 4 consecutive backcrossing generations, we then used MAS to identify individual plants with the genotype *RrMs_3_ms_3_Rf^b^Rf^c^*, ultimately selecting 9 individuals from this BC_4_F_1_ population. Subsequently, a comparative analysis of the genetic backgrounds was performed between these 9 plants and the original parent YA001 using the SNP panel technology. The results revealed that a genetic similarity coefficient arranged from 0.912 to 0.947 of these 9 plants to the recurrent parent (**Supplementary Table 1**), indicating that through 4 generations of backcross breeding, the highest recurrent rate has successfully retained 94.7% of the genetic background. Then, these 9 plants were bagged for self-pollination to generate BC_4_F_2_ population, respectively. Among them, the selfed seeds line named as MR105 were sown and leaf samples were collected at the seedling stage for DNA extraction and genotyping. Line MR105 produced 204 selfed progeny plants. From these, 8 homozygous disease-resistant target plants were selected (**Table 3**), including two homozygous disease-resistant temporary maintainer lines (*RRms_3_ms_3_Rf^c^Rf^c^*), four fertile homozygous disease-resistant two-type lines (*RRMs_3_ms_3_Rf^b^Rf^b^*) and two sterile homozygous disease-resistant two-type lines (*RRms_3_ms_3_Rf^b^Rf^b^*). The progeny of *RrMs_3_ms_3_Rf^b^Rf^c^* genotype would segregate into 27 possible genotypic combinations, the probability of obtaining each of the three target genotypes we required is 1/64. Therefore, to ensure the selection of all target genotypes and secure viable seeds, it is advisable to have at least 5 individuals per selected genotype. This necessitates a population of over 300 individuals in the self-pollinated progeny.

**Table 3:** Homozygous CR Genic Male Sterile three-line system plants were selected from BC_4_F_2_ of line MR105.

Plant number	Generation	Genotype	Type
MR105-18	BC_4_F_2_	*RRms_3_ms_3_Rf^c^Rf^c^*	Homozygous CR TM line
MR105-84	BC_4_F_2_	*RRms_3_ms_3_Rf^c^Rf^c^*	Homozygous CR TM line
MR105-4	BC_4_F_2_	*RRMs_3_ms_3_Rf^b^Rf^b^*	Homozygous CR B line
MR105-195	BC_4_F_2_	*RRMs_3_ms_3_Rf^b^Rf^b^*	Homozygous CR B line
MR105-33	BC_4_F_2_	*RRms_3_ms_3_Rf^b^Rf^b^*	Homozygous CR A line
MR105-48	BC_4_F_2_	*RRms_3_ms_3_Rf^b^Rf^b^*	Homozygous CR A line
MR105-147	BC_4_F_2_	*RRms_3_ms_3_Rf^b^Rf^b^*	Homozygous CR A line
MR105-202	BC_4_F_2_	*RRms_3_ms_3_Rf^b^Rf^b^*	Homozygous CR A line

Next, two selected homozygous TM lines were bagged for self-pollination and propagation. Meanwhile, the 6 homozygous resistant TT lines including fertile and sterile plants as shown in **Table 3** above were transplanted together and cross to each other when flowering, and then seeds from the sterile plants were harvested to propagate the two-type line.

### 3.3 Inoculation identification of advanced-generation clubroot-resistant lines

Due to the limited number of selected plants in each backcross generation and the need for subsequent backcrossing, disease resistance screening was not conducted immediately in the backcrossed generation. Instead, molecular marker selection was performed first, with indoor pathogen identification carried out at advanced generations.

Upon reaching the BC_4_F_2_ generation, two self-pollinated lines MR105–45 and MR105–63 were selected based on molecular marker results for indoor artificial clubroot inoculation identification. Their parental genotypes were: MR105-45 (*Rr* heterozygous resistant), MR105-63 (*RR* homozygous resistant), and a susceptible TM line (*rr*) without resistance genes served as the control. The indoor inoculation identification results are shown in **Table 4**.

**Table 4:** Disease statistics of indoor inoculation.

Sample	Grading				Disease incidence rate(%)	Disease index
	0 grade	1 grade	2grade	3grade		
susceptible control(*rr*)	1	10	8	28	97.9%	78.0
MR105-45(*Rr*)	18	11	9	10	62.5%	41.0
MR105-63(*RR*)	39	3	3	1	15.2%	8.7

**Figure 3:**
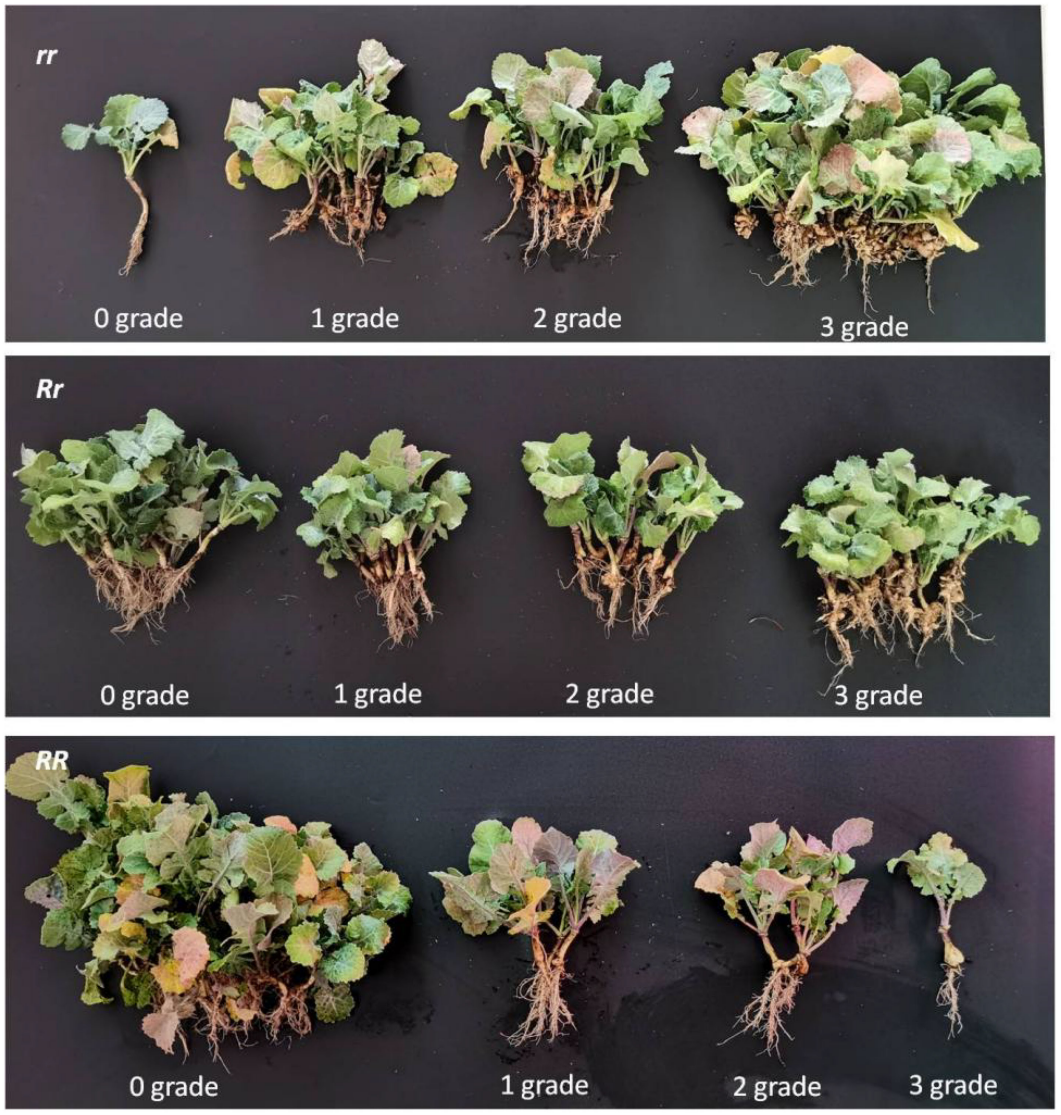
The incidence statistics of different genotypes with strains indoor inoculation. *RR* represents homozygous genotype, *Rr* represents heterozygous genotype and *rr* represents susceptible genotype for resistance.

As shown in **Table 4**, the susceptible control exhibited a disease incidence rate of 97.9% with a disease index of 78, indicating high susceptibility. In contrast, the homozygous resistant line MR105–63 showed significantly reduced disease incidence, with only one plant rated as grade 3 susceptible and a disease index of 8.7, demonstrating high resistance. The heterozygous resistant line MR105–45 displayed higher disease incidence than the homozygous resistant line but lower than the susceptible control, which aligns with expectations since it segregates 1/4 susceptible genotypes (**Figure 3**).

### 3.4 Seed production and hybrid combination of clubroot-resistant three-line system

**Figure 4:**
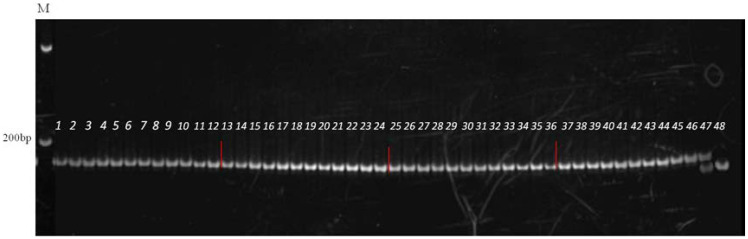
The testing results of homozygous resistant lines and all individuals showed RR genotypes. Lane 1–46 were testing plants and lane 47and 48 were the *Rr/rr* control genotype, respectively. M: DNA marker.

After scaling up seed production of CR temporary maintainer line and two-type lines, 46 randomly selected plants were genotyped using molecular markers to verify resistance genotypes and prevent potential field contamination or resistant gene loss. All tested plants were confirmed to maintain homozygous resistant genotypes (**Figure 4**).

**Figure 5:**
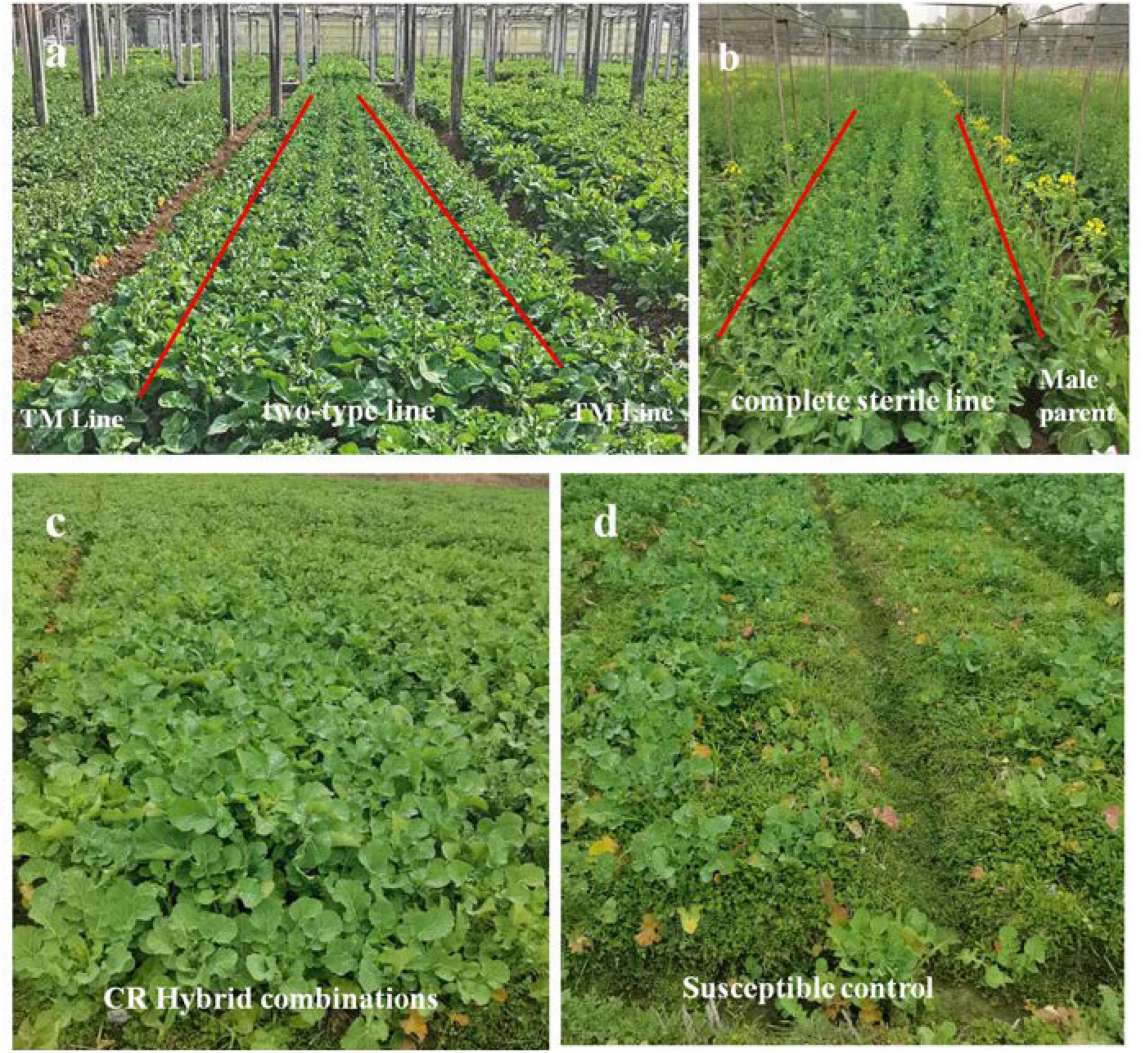
Seed production and field performance of clubroot-resistant Hybrid Combinations. **(a)** shows production of complete sterile lines, where two-type line and TM line were planted in a 4:2 row ratio; **(b)** shows CR hybrid combinations producing, CR completely sterile lines were used as the female parent and 11 elite inbred lines as male parents, and they were planted in a 4:2 row ratio; **(c)** shows field performance of CR hybrid combinations in the clubroot-endemic region; **(d)** shows field performance of susceptible control ‘Fengyou 737’ in the clubroot-endemic region.

Then complete sterile lines would be produced for hybrid combination. The two-type lines and temporary maintainer lines were planted in a 4:2 row ratio in the field (**Figure 5a**). During flowering, pollen fertility survey was conducted and 50% of fertile plants were removed from the two-type line population and then pollination tents were set up with bees for pollination. To prevent mixing, TM lines were cut after flowering. At maturity, seeds were harvested from sterile plants and these seeds were complete sterile lines, which were then used to develop hybrid combinations.

### 3.5 Field performance of clubroot-resistant hybrid combinations

Hybrid combinations were developed using the CR completely sterile lines as the female parent and 11 elite inbred lines (restorer lines) as male parents, and they were planted in a 4:2 row ratio in the field too (**Figure 5b**). The F1 hybrid seeds were harvested from the female (CS) plants following their crossing with restorer lines.Then F_1_ hybrid combinations were cultivated in the clubroot-endemic area of Xiuning County, Huangshan City (where physiological race 4 is the predominant pathotype), with the susceptible hybrid ‘Fengyou 737’serving as the control. All experimental plots were arranged in a completely randomized design with three replications. Disease incidence of clubroot at seedling stage and agronomic traits at harvest stage were evaluated.

The seedling-stage clubroot assessment revealed that the susceptible control ‘Fengyou 737’showed severe infection, with a disease incidence rate reaching 78.3% and seedling mortality exceeding 50%, indicating high susceptibility (**Table 5**, **Figure 5c**). In contrast, all 11 hybrid combinations derived from YA001R exhibited mild symptoms with only sporadic infected plants, demonstrating disease indices below 10 and qualifying as highly resistant.

**Table 5:** Evaluation form for traits of YA001R hybrid combinations cultivated in clubroot disease epidemic areas.

Hybrid combinations	Plant height (cm)	Productive branch position (cm)	Number of primary branches	Number of siliques per plant	Seeds per silique	1000-seed weight (g)	Seed yield (kg/ha)	Incidence rate (%)	Disease Index	Yield ranking
PV01	149.5	51.0	7.0	277.7	22.0	5.51	3020.1	9.3	5.8	6
PV02	152.8	70.6	8.7	296.9	21.0	4.71	2998.5	12.1	6.7	7
PV03	149.5	58.3	6.3	272.0	22.2	5.86	3141.6	11.0	6.5	3
PV04	143.9	62.7	9.3	286.7	21.0	5.15	2833.2	12.2	6.7	9
PV05	155.2	67.0	8.3	270.6	19.3	5.30	3161.7	9.7	5.9	2
PV06	150.7	59.3	5.7	266.7	18.2	5.65	2397.9	7.8	4.7	11
PV07	145.9	64.3	9.0	304.2	18.4	4.75	3273.15	6.9	5.3	1
PV08	145.6	72.6	6.7	276.0	21.1	4.72	3096.6	5.3	5.3	5
PV09	136.8	62.4	7.0	294.2	16.5	5.35	2815.2	7.2	6.1	10
PV10	147.9	69.7	8.3	299.4	17.2	4.85	3138.15	4.7	2.3	4
PV11	150.0	71.7	8.7	291.2	20.7	5.56	2965.2	5.3	3.4	8
PV12(CK)	135.5	52.5	8.7	136.3	15.7	3.33	1279.8	87.3	74	12

Regarding yield performance, the susceptible control suffered substantial seedling mortality, resulting in significantly reduced final yield (1279.8 kg/ha). The highest-yielding resistant combination achieved 3273.15 kg/ha. Compared to the resistant hybrids, the susceptible control also displayed markedly inferior agronomic traits, including plant height, effective siliques per plant, and 1000-seed weight.

## 4 Discussion

The recessive genic male sterility (RGMS) three-line system in *Brassica napus* has been utilized in hybrid production for over two decades. However, due to its complex genetic relationships, many field breeders still develop the TT line and the TM line through separate hybridization and test-crossing breeding processes. As a result, the selected 3 lines possess different genetic backgrounds, leading to the production of hybrid seeds that are essentially triple-cross hybrids. This not only results in poor uniformity of the hybrids but also reduces hrbrid heterosis.

This study employed molecular markers to simultaneously screen for the clubroot resistance gene *CRb* and fertility-related genes *Ms_3_/ms_3_* and *Rf^b^/Rf^c^*. Through backcrossing and selfing, with each backcrossing generation selecting for the *RrMs_3_ms_3_Rf^b^Rf^c^* genotype, a homogeneous CR TM line and TT lines were successfully developed. Since all lines derived from the same parental line, they share an identical genetic background. We further performed SNP panel to evaluate the genetic distance between the A and TM lines. The resulting genetic similarity coefficient was 0.947, indicating high similarity. Field observations also showed they exhibit highly consistent agronomic traits (**Figure 5a**). Consequently, the hybrid F_1_ demonstrated uniform performance in the field, effectively resolved the problem of declined heterosis of triple-cross hybrids.

The integration of background selection may significantly accelerate the breeding process. Li used background and foreground selection and obtained plants with over 97% genetic background recovery at BC_3_ generation (Li et al., 2021). In this study, background selection was not implemented, which necessitated larger population sizes and increased labor and cost input. Nevertheless, by selecting individuals with superior agronomic traits through field observation, approximately 95% recovery of the genetic background was achieved after 4 backcross generations. This demonstrates that BC_4_ alone can largely fulfill the requirements of backcross breeding programs.

Hybrid combinations derived from YA001R demonstrated excellent clubroot resistance and superior yield performance. Li (Li et al., 2021) reported that the donor parent ‘Bing409R’ demonstrated immune-level resistance to *P. brassicae* isolates collected from Yichang and Zhijiang (Hubei Province), Huangshan (Anhui Province), and various regions in Sichuan Province, indicating that the developed RGMS line YA001R possesses substantial breeding potential and application prospects. However, due to the high diversity of *P. brassicae* in China and the prevalent coexistence of multiple pathotypes in field populations, single-gene-mediated resistance is vulnerable to breakdown through shifts in dominant physiological races (Cao et al., 2009). Studies by Shah and Baloch revealed that homozygous resistant genotypes exhibit stronger resistance than heterozygous, and pyramiding two resistance genes provides broader-spectrum protection compared to single-gene resistance (Shah et al, 2019; Baloch et al 2024). This evidence confirms that gene pyramiding facilitates the development of durably resistant cultivars. Accordingly, the integration of two resistance genes (*PbBa8.1* + *CRb*) into the YA001 has progressed to the BC_4_ generation. These improved genetic resources will constitute a crucial germplasm foundation for controlling the spread of clubroot disease.

The research also provides a simple, rapid, and efficient breeding method for obtaining elite *Brassica napus* clubroot-resistant homogeneous three-line systems. Using this approach, any elite parental line can be modified and converted into a clubroot-resistant homogeneous genic male sterile three-line system. The study establishes a material foundation and provides technical support for ensuring the production security of the rapeseed industry.

## Data Availability

The original contributions presented in the study are included in the article/**Supplementary Material**. Further inquiries can be directed to the corresponding author.
