# Supplementary material for: Genetic improvement of clubroot resistance in the recessive genic male sterile line YA001 in Brassica napus
Source: Front Plant Sci. 2025 Dec 16;16:1726940. doi: 10.3389/fpls.2025.1726940 (PMC12748198; doi:10.3389/fpls.2025.1726940)
Supplement: Supplementary file 1 [file Table1.docx]

Supplemental table 1 The geno-similarity coefficients between YA001 and the 9 plants

|  | YA001 | 1 | 2 | 3 | 4 | 5 | 6 | 7 | 8 | 9 |
| --- | --- | --- | --- | --- | --- | --- | --- | --- | --- | --- |
| YA001 | 1 | 0.921 | 0.927 | 0.936 | 0.944 | 0.912 | 0.932 | 0.947 | 0.926 | 0.933 |
| 1 |  | 1 | 0.917 | 0.922 | 0.923 | 0.913 | 0.931 | 0.936 | 0.914 | 0.926 |
| 2 |  |  | 1 | 0.931 | 0.929 | 0.921 | 0.939 | 0.931 | 0.925 | 0.931 |
| 3 |  |  |  | 1 | 0.932 | 0.918 | 0.944 | 0.941 | 0.933 | 0.929 |
| 4 |  |  |  |  | 1 | 0.922 | 0.932 | 0.955 | 0.942 | 0.952 |
| 5 |  |  |  |  |  | 1 | 0.928 | 0.921 | 0.923 | 0.922 |
| 6 |  |  |  |  |  |  | 1 | 0.939 | 0.93 | 0.943 |
| 7 |  |  |  |  |  |  |  | 1 | 0.951 | 0.955 |
| 8 |  |  |  |  |  |  |  |  | 1 | 0.934 |
| 9 |  |  |  |  |  |  |  |  |  | 1 |
